# Supplementary material for: Electronic control of gene expression and cell behaviour in Escherichia coli through redox signalling
Source: Nat Commun. 2017 Jan 17;8:14030. doi: 10.1038/ncomms14030 (PMC5247576; doi:10.1038/ncomms14030)
Supplement: Supplementary Information — Supplementary Figures, Supplementary Tables, Supplementary Notes, and Supplementary References [file ncomms14030-s1.pdf]

Supplementary Information

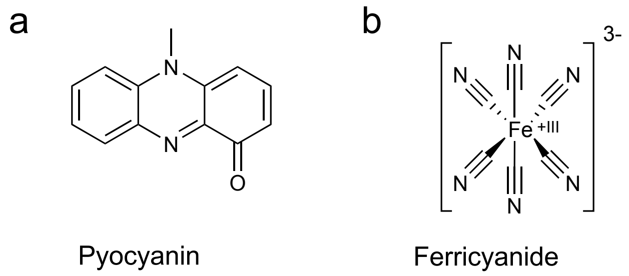

Supplementary Figure 1. Redox mediator structures: a. The chemical structure of pyocyanin. **b.** The chemical structure of ferricyanide.

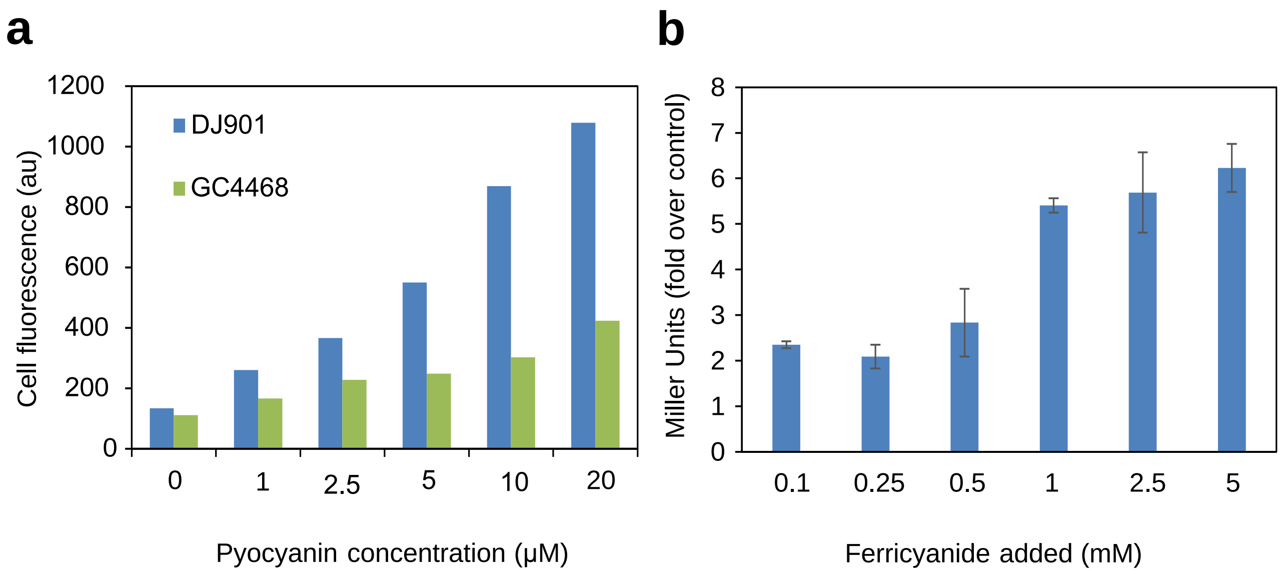

Supplementary Figure 2. Response of cells with intact *soxRS* : a. phiLOV fluorescence of GC4468 or DJ901 (GC4468 *ΔsoxRS)* cells induced anaerobically with the indicated pyocyanin concentrations for 1 hour. **b.** Miller Units resulting from pyocyanin (2.5 µM) and ferricyanide (concentrations indicated) treatment of ZK126 cells with the pTG1 plasmid anaerobically for 1 hour. Pyocyanin-only control was 153 MU. Error bars indicate s.d. of biological duplicates.

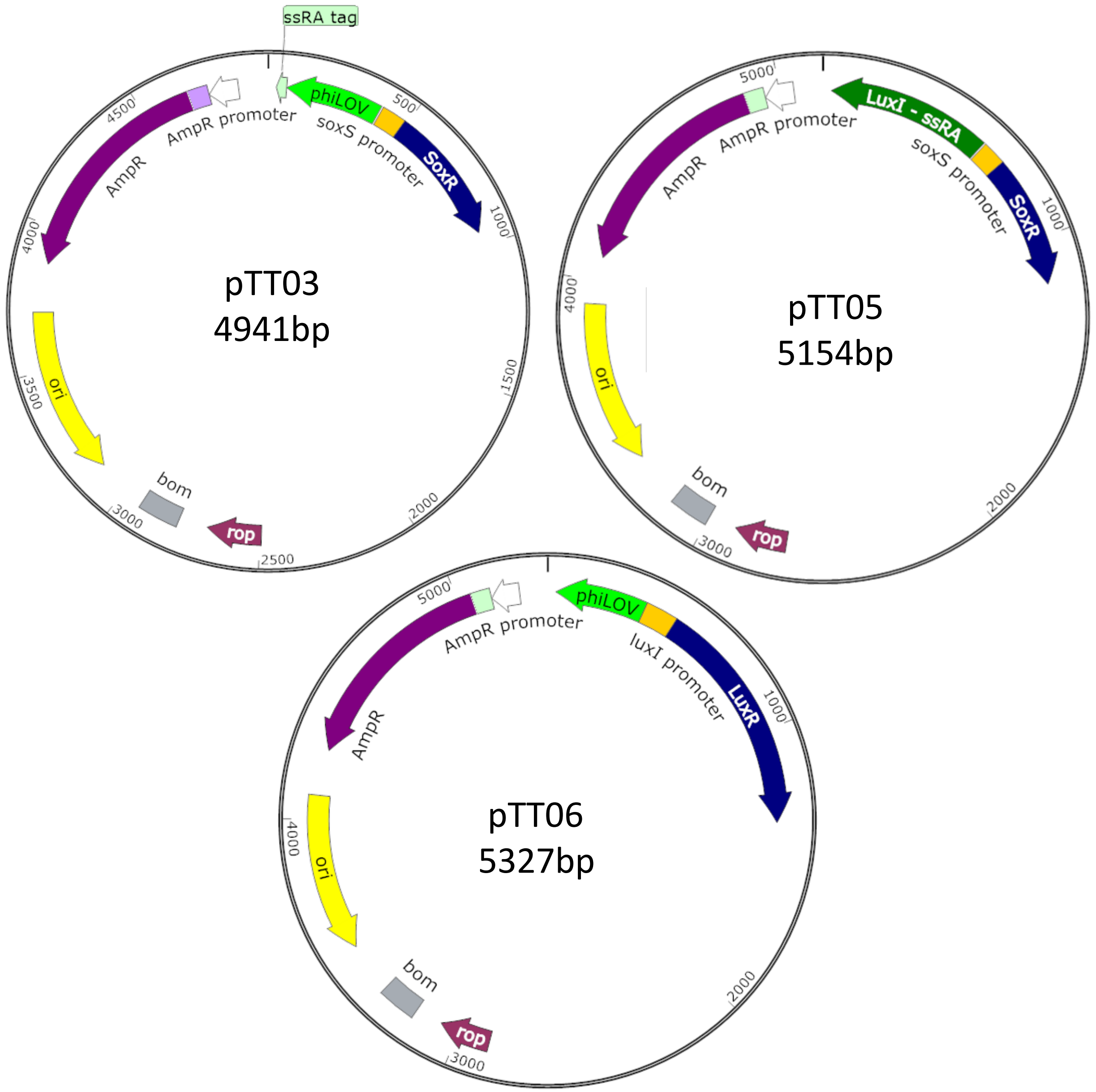

Supplementary Figure 3. Plasmid maps: Maps of the pBR322-based plasmids that were predominately used in this work. ssRA tag denotes either the LAA or DAS tag.

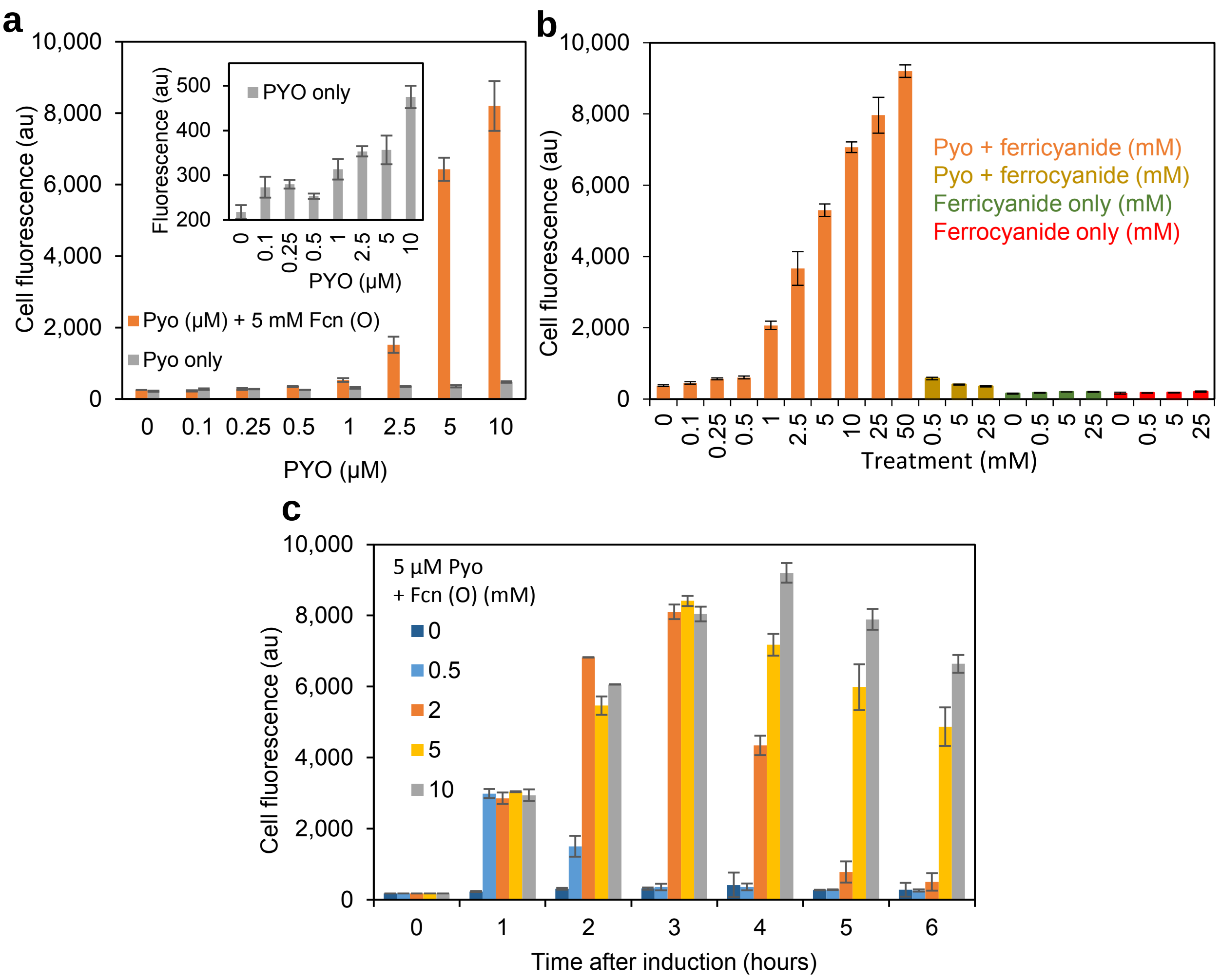

Supplementary Figure 4. Mediator effects on induction from Ps*oxS* promoter: a. Fluorescent protein induction due to pyocyanin with or without the addition of ferricyanide. **b.** Fluorescent protein induction due to varying concentrations of ferricyanide or ferrocyanide added with or without 5 μM pyocyanin. **c.** Fluorescent protein induction time profile with the same pyocyanin but different ferriycanide amounts. Error bars indicate s.d. of biological triplicates.

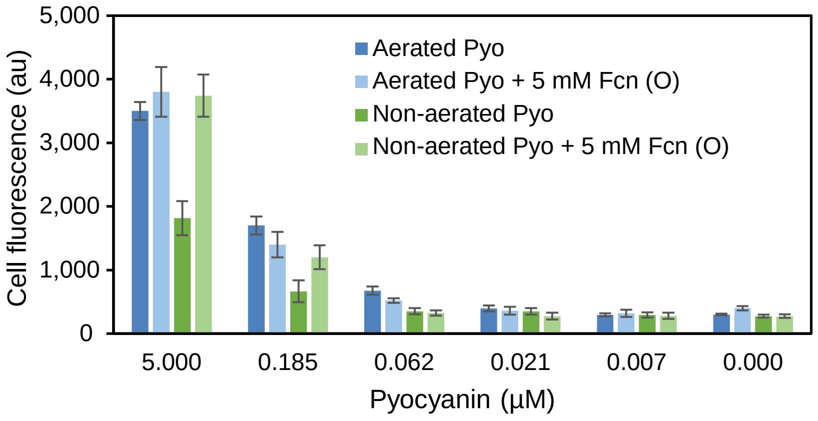

Supplementary Figure 5. Induction of fluorescence in aerobic conditions: Cell fluorescence measured after induction with different concentrations of pyocyanin with or without 5 mM ferricyanide (Fcn (O)) in aerobic conditions. Cultures were either aerated (at 250 rpm) or non-aerated (stationary). Error bars represent s.d. of biological duplicates.

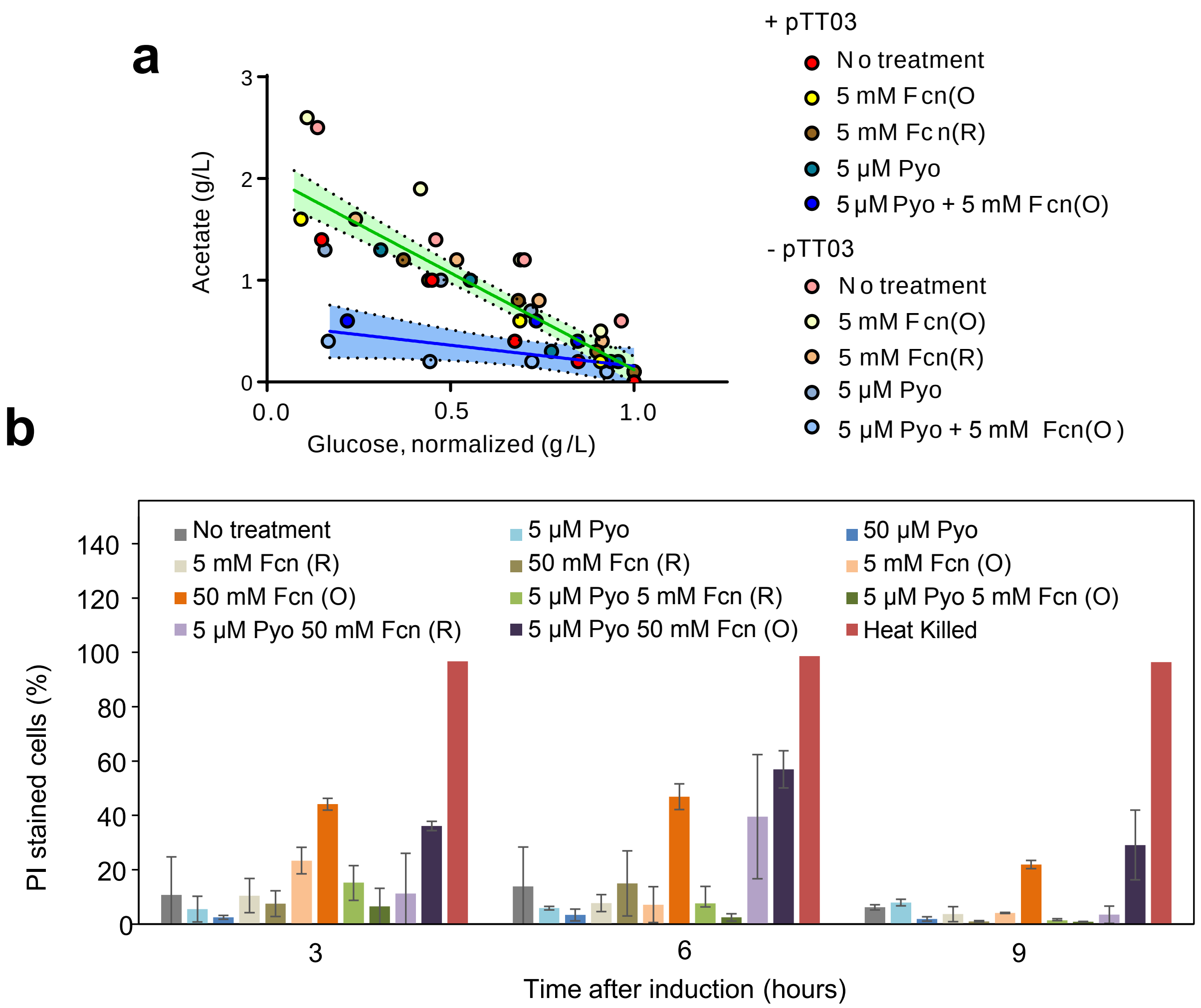

Supplementary Figure 6. Metabolic effects and propidium iodide analysis: a. Glucose consumed (normalized to initial measurement) plotted vs. the acetate produced for DJ901 cells with or without the pTT03 plasmid treated with the indicated mediators. Green and blue shaded regions represent 95% confidence intervals for green and blue trendlines, respectively. **b.** The percent of DJ901 pTT03 cells stained with propidium iodide, indicating dead cells. Error bars show s.d. of biological triplicates.

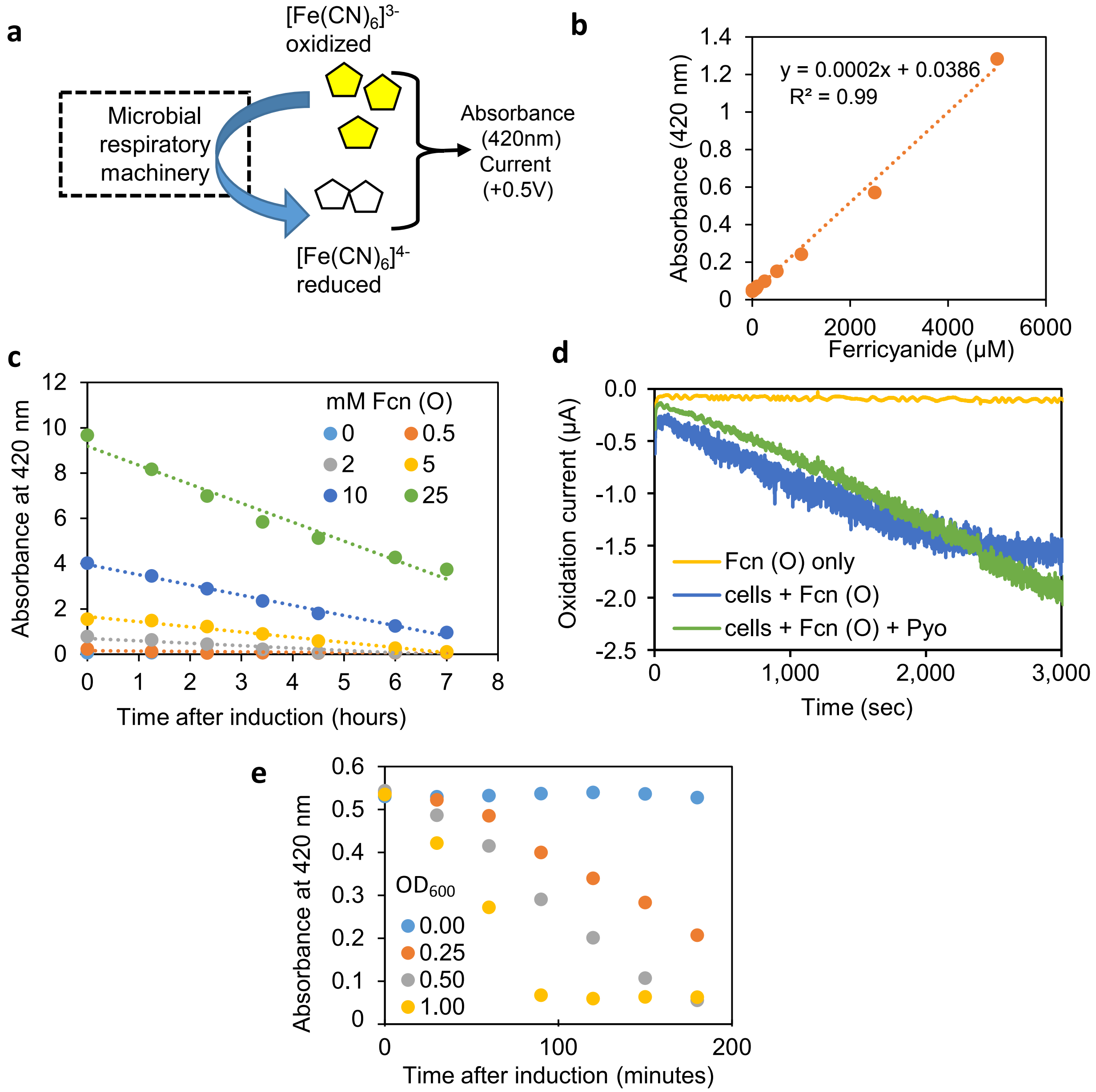

Supplementary Figure 7. Cell reduction of ferricyanide: a. Scheme of spectrophotometric and electrochemical methods for measuring ferricyanide reduction by cells. **b**. Absorbance at 420 nm correlates with ferricyanide concentration. **c.** Reduction of different ferricyanide concentrations by cells results in absorbance decrease (measured at 420 nm, starting cell OD_{600} of 2.0) over time. **d.** Oxidation of ferrocyanide shows more negative current (measuring ferrocyanide) when ferricyanide is reduced by cells. **e.** Reduction of ferricyanide, as measured by absorbance, is higher with higher cell amounts.

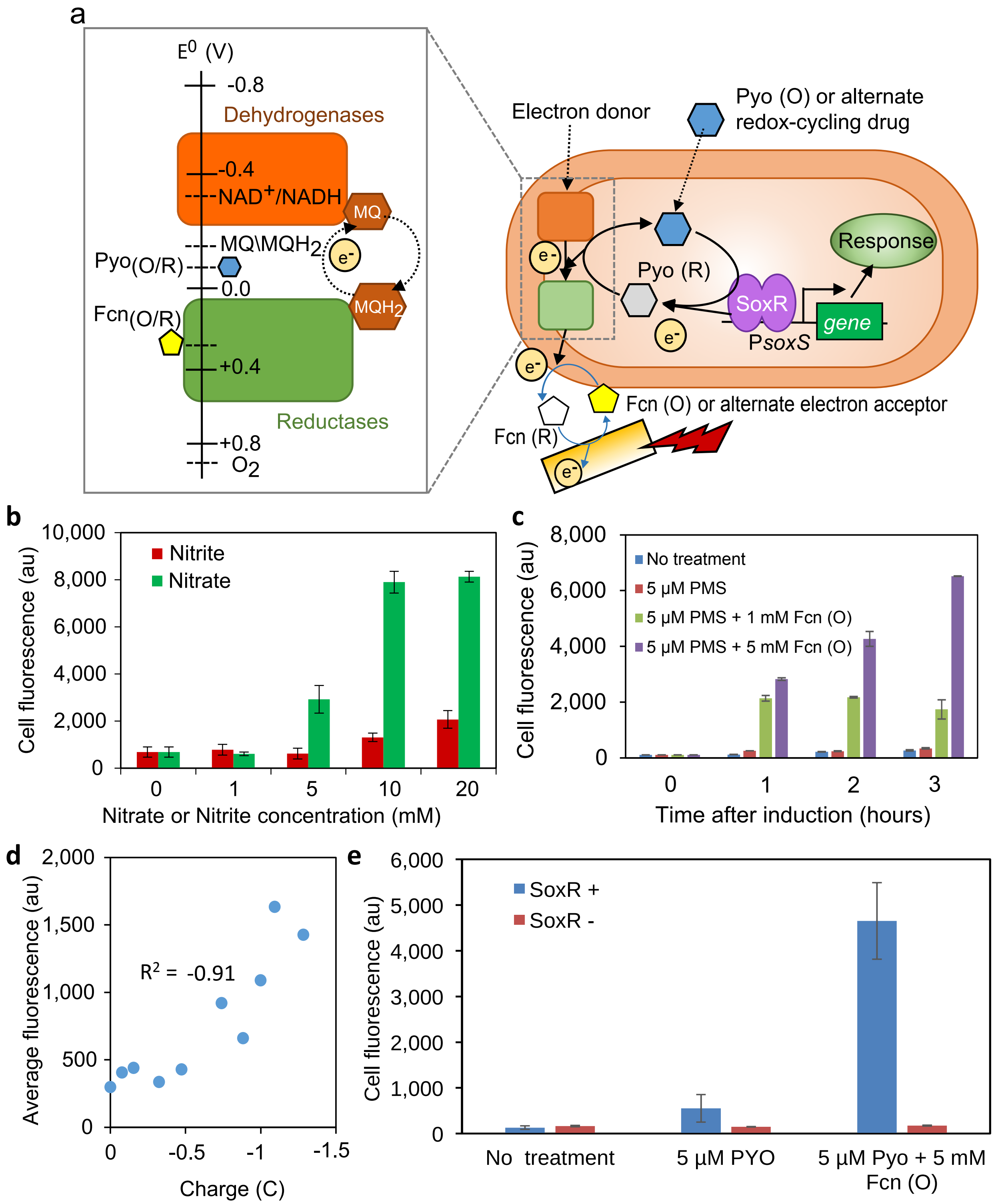

Supplementary Figure 8. Effect of alternate redox mediators on cell response: a. Schematic of

potential mechanism of ferricyanide-based pyocyanin-driven gene induction amplification from the P*soxS* promoter. **b.** Fluorescence of cells after 1.5 hours, producing the phiLOV protein in response to 5 µM added pyocyanin and the indicated concentrations of either nitrate or nitrite. 5 µM Na Myolobdate was added to all samples. **c.** Cell fluorescence over time in response to addition of PMS with or without Fcn (O). **d.** Electronic control of cell fluorescence with charge with 5 mM Fcn (R) and 5 µM PMS. **e.** Fluorescence of DJ901 cells with or without the *soxR* gene on the plasmid in addition to the P*soxS* promoter and *phiLOV* gene induced with the indicated mediators. Error bars indicate standard deviation of biological duplicates in b and c and triplicates in e. R^2 indicates Spearman correlation coefficient for monotonic correlation.

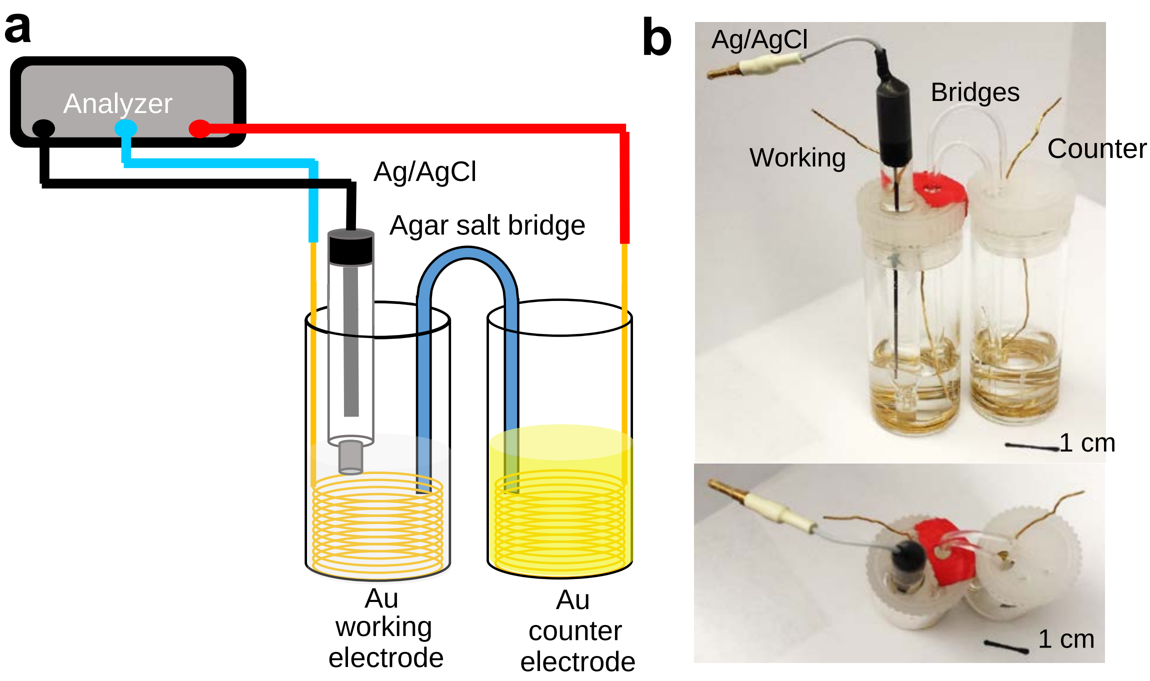

Supplementary Figure 9. Electrochemical bulk electrolysis setup: a. An electrochemical analyzer is connected to a computer and three electrodes – gold (Au) working and counter electrodes and an Ag/AgCl reference electrode. The working and counter electrodes are separated by two agar salt bridges. **b.** Photographs of setup used for bulk electrolysis and *in situ* electrochemical cell induction. Liquid level indicates 3 mL of solution.

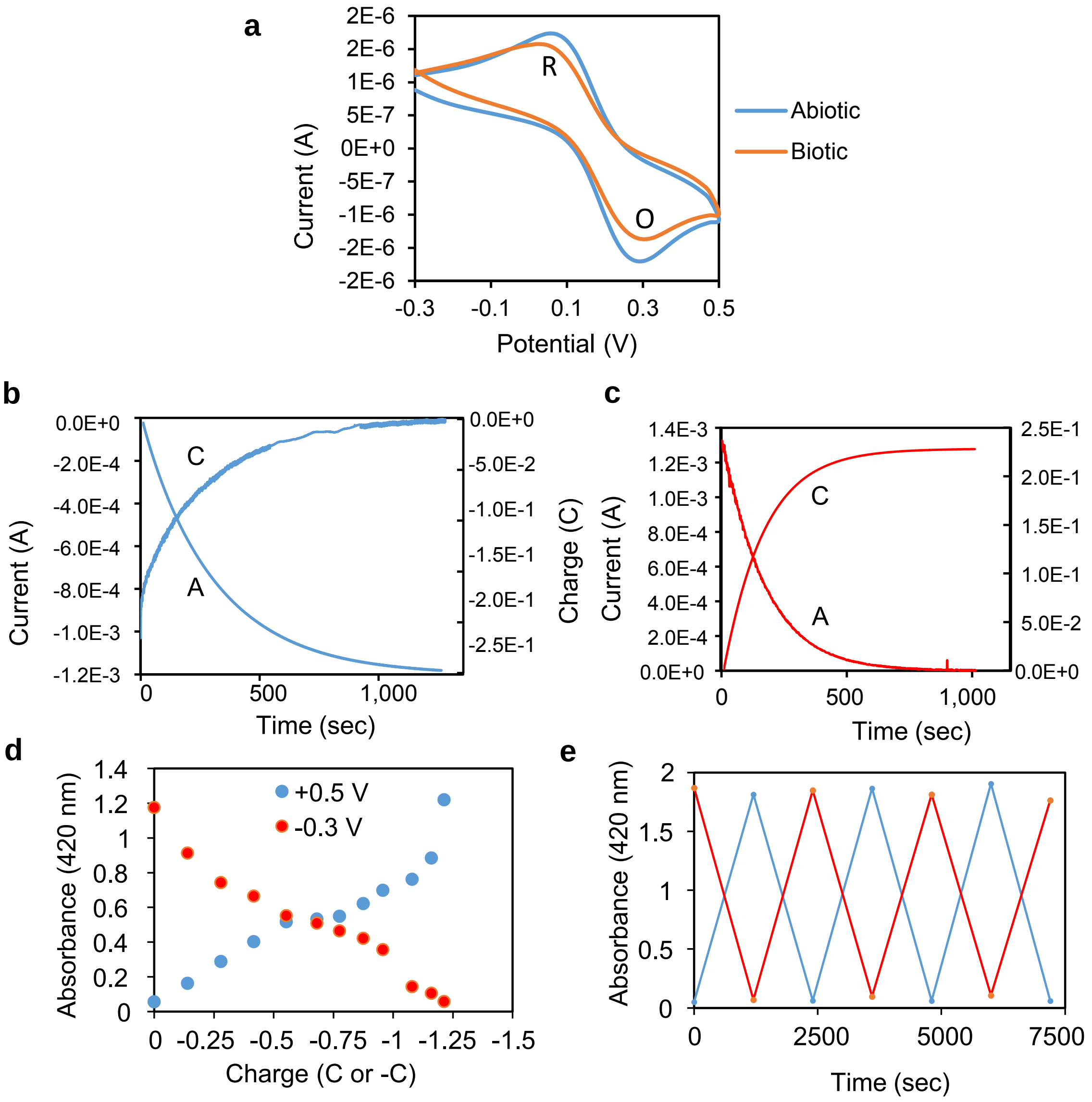

Supplementary Figure 10. Electrochemical ferro/ferricyanide redox-state interconversion: a. Sample cyclic voltammograms of 0.5 mM ferri/ferrocyanide indicating the reduction (R) and oxidation (O) peaks. Biotic sample included cells at OD600 at 0.25. **b.** Current and charge over time of bulk oxidation of 5 mM Fcn (R). **c.** Current and charge over time of bulk reduction of 5 mM Fcn (O). **d.** Absorbance change with charge as Fcn (R) is oxidized (+0.5 V) or Fcn (O) is reduced (-0.3 V). **e.** Multiple cycles of oxidation/reduction on the same solution and corresponding changes in absorbance over time.

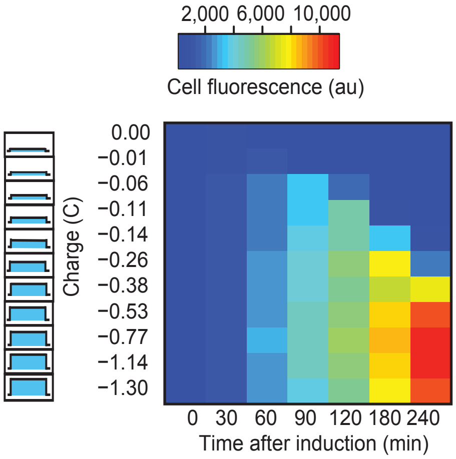

Supplementary Figure 11. Cell fluorescence due to varying voltages: Heat map depicting cell fluorescence over time in response to application of various voltages (from Figure 2 b) for 15 minutes, then continued cell response and sampling.

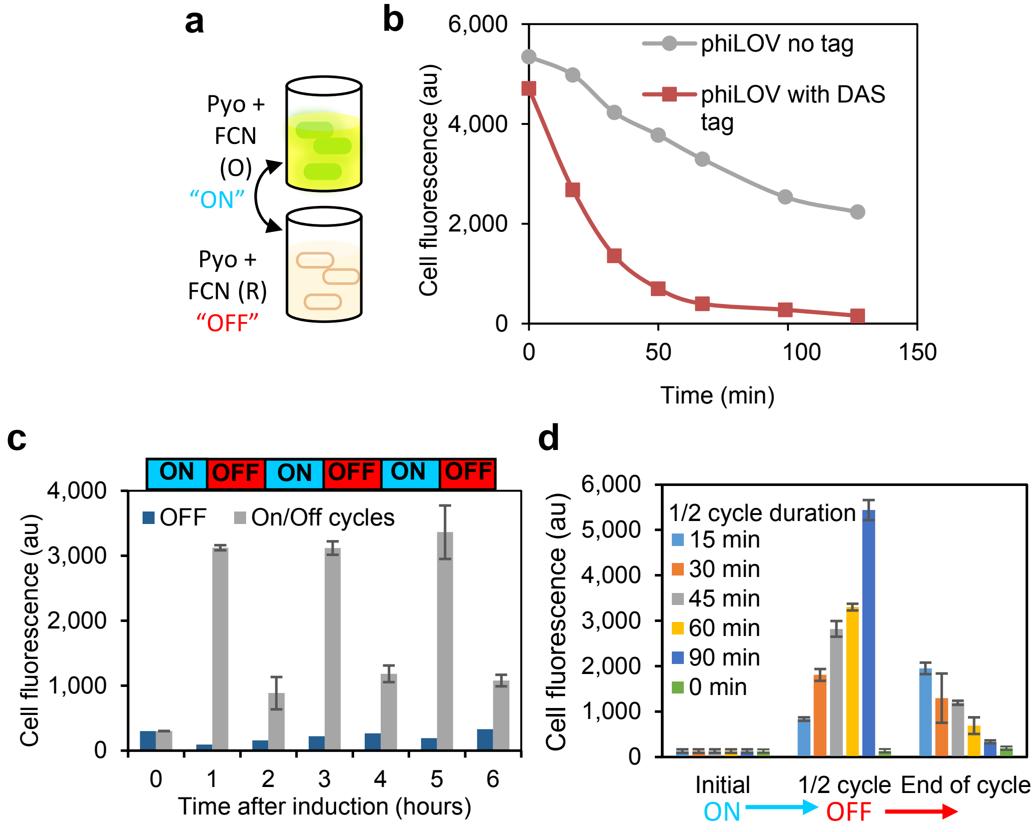

Supplementary Figure 12. Ferricyanide and ferrocyanide control of protein levels: a. Schematic of experiments in c and d where cells are first induced with pyocyanin and Fcn (O) for a ½ cycle duration ("ON"), then spun down and re-suspended in pyocyanin with Fcn (R) ("OFF"). **b.** Cell fluorescence degradation of phiLOV with the DAS tag or phiLOV without a tag. Time is after re-suspension in fresh media. **c.** Repeated "ON"/"OFF" cycles show that cells continue to respond over time. **d.** A single "ON"/"OFF" cycle of various half-cycle lengths shows cell response time to changing Fcn(O) to Fcn(R). Error bars indicate standard deviation of biological triplicates.

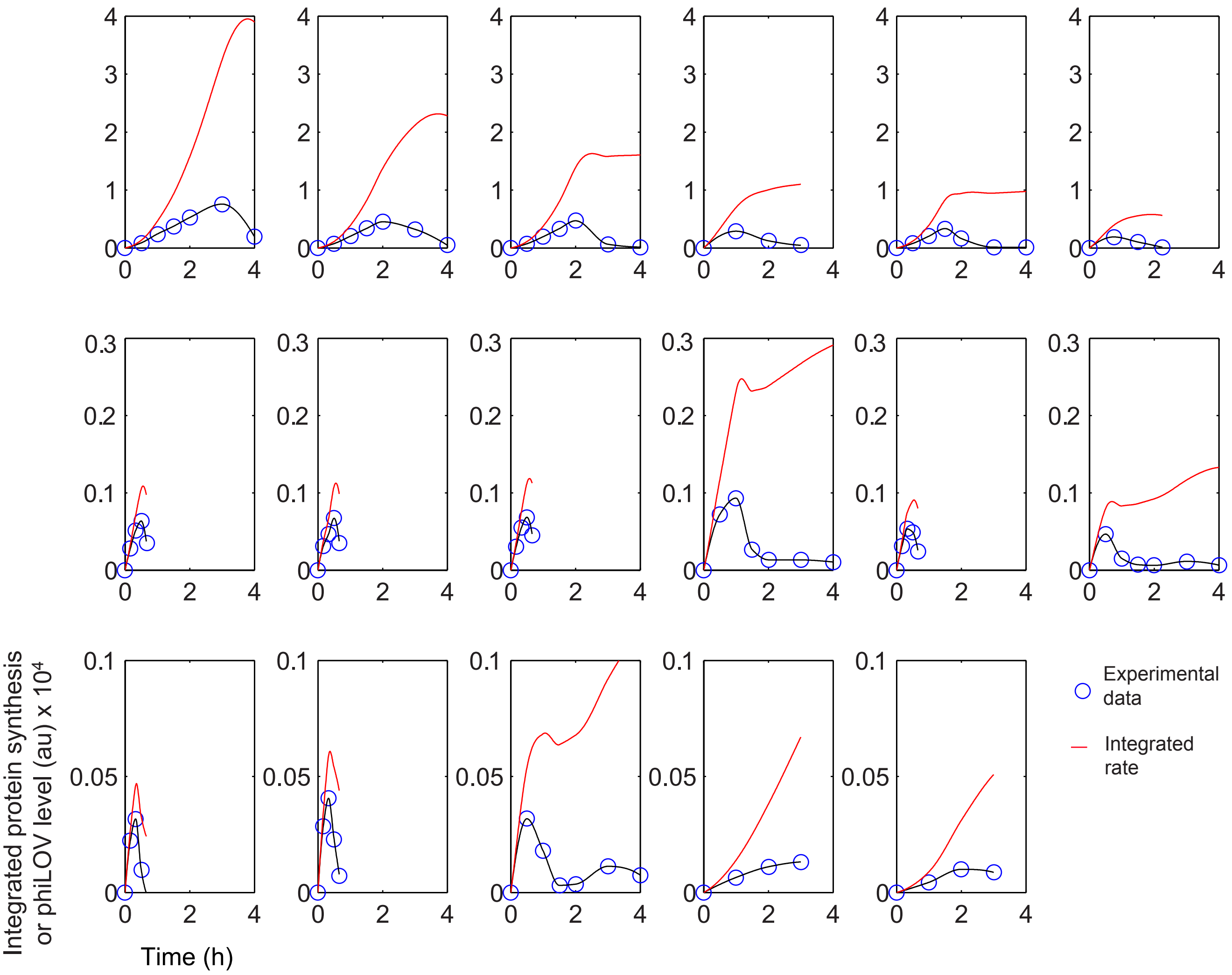

Supplementary Figure 13. Model results. Red line indicates integrated rate (from model). Blue circles with black line represents experimental data.

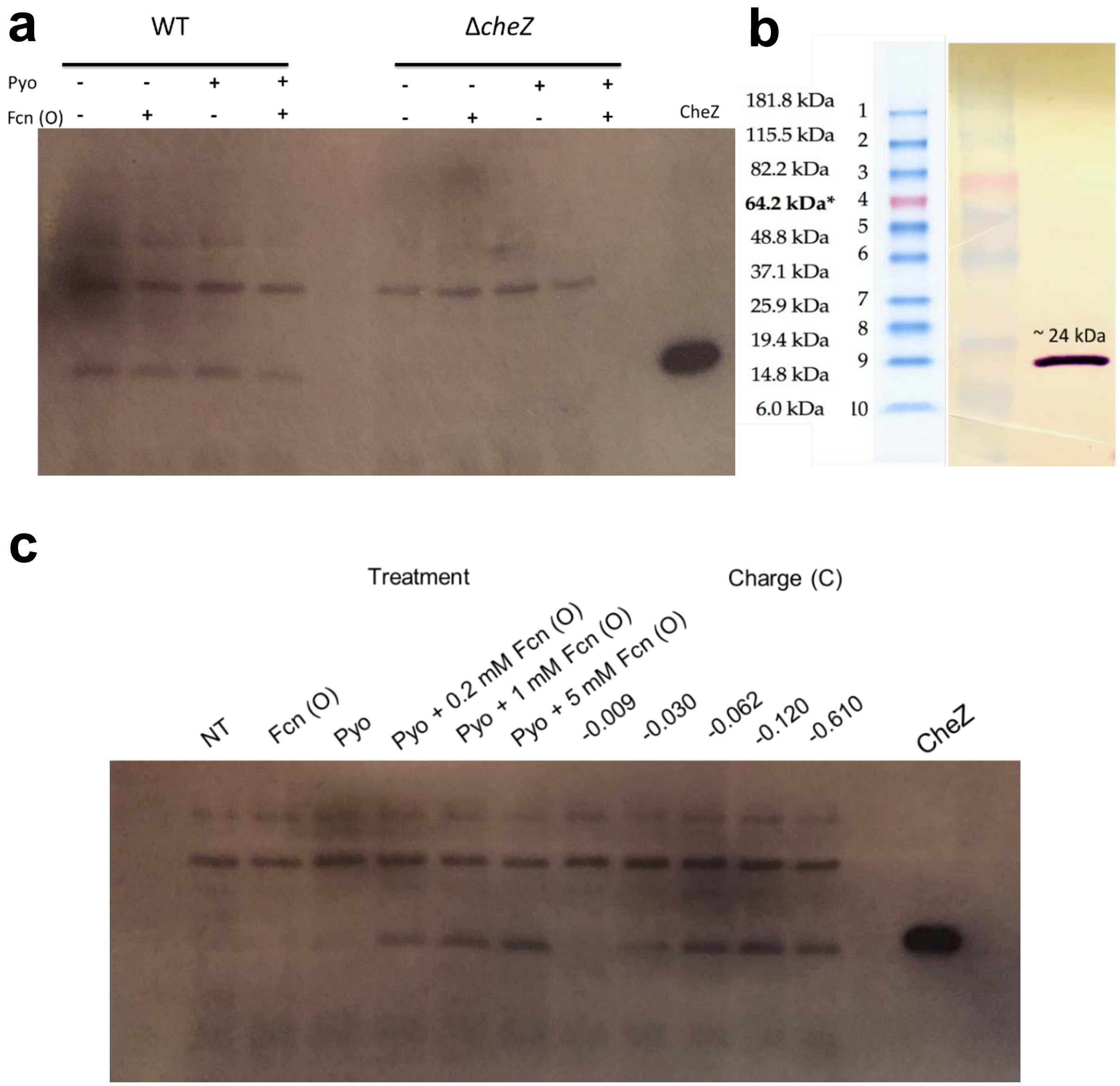

Supplementary Figure 14. CheZ controls Western blots: a. Western blots of the CheZ protein of WT and CheZ KO cells treated with the indicated mediators. Pyocyanin was 5 μM and Fcn (O) was 5 mM. **b.** Ladder used for CheZ size determination, which is about 24 kDa. **c.** Western blot as seen in Figure 4 b, uncropped. Fcn (O), when not indicated, was at 5 mM. Pyocyanin was 5 μM. Charges applied as indicated in Methods. NT indicates no treatment.

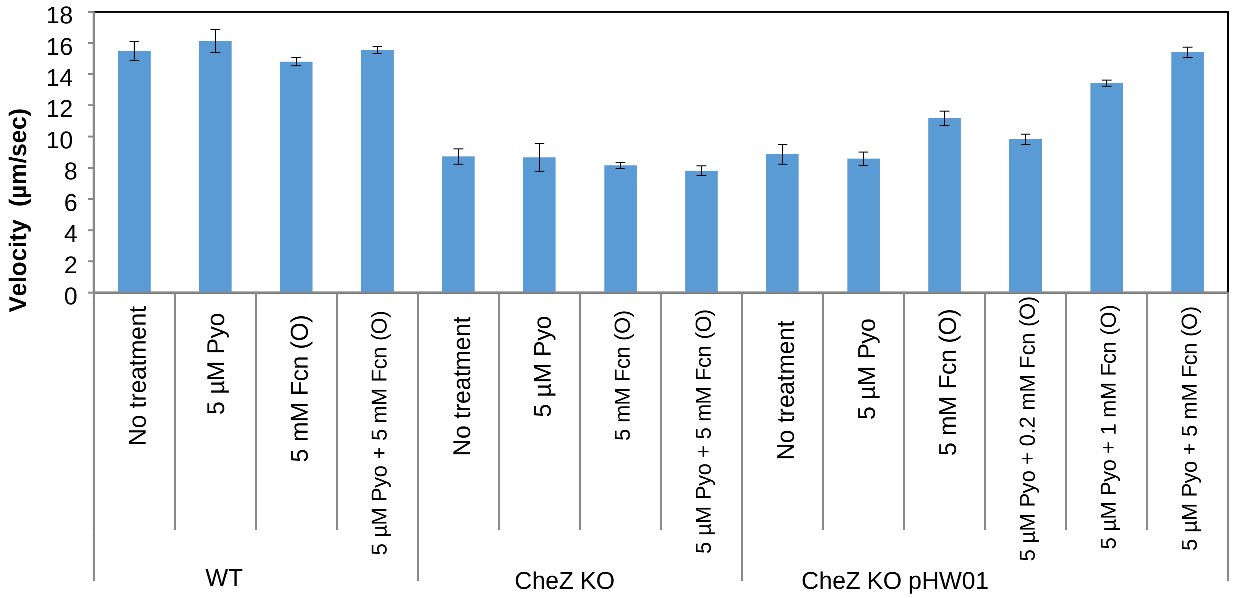

Supplementary Figure 15. Cell velocities: Cell velocities of various cells treated with the mediators in the indicated concentrations. Error bars represent standard error of 50 – 800 separate cell trajectories per sample.

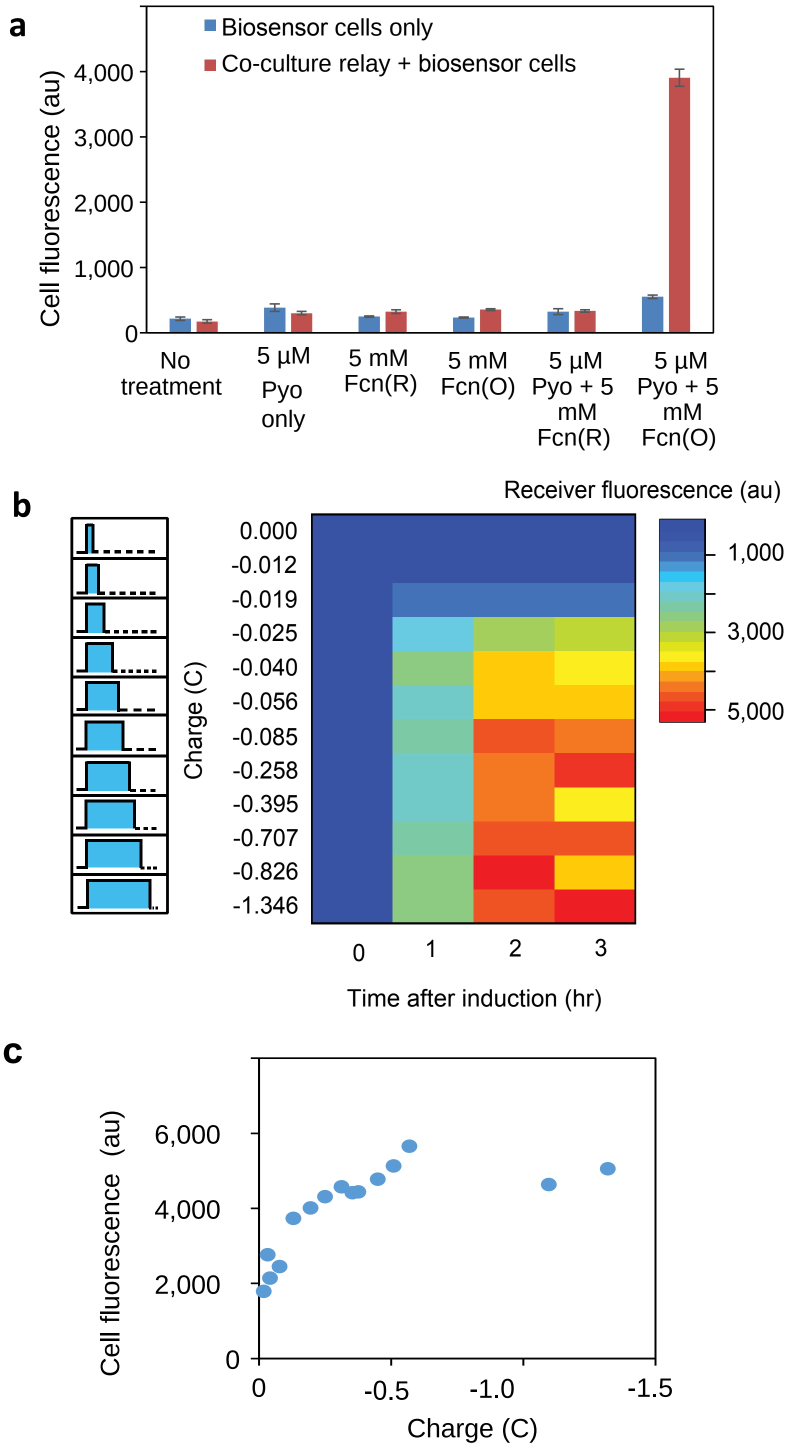

Supplementary Figure 16. AHL solution-based induction: a. Fluorescence of either the reporter cells only or co-culture of reporter and relay cells in response to the indicated mediators. **b.** Fluorescence over time of reporter cells in co-cultures induced with the indicated charges. **c.** Fluorescence of the reporter cells when treated with supernatants of the relay cells induced with the indicated charges. Error bars indicate s.d. of biological triplicates.

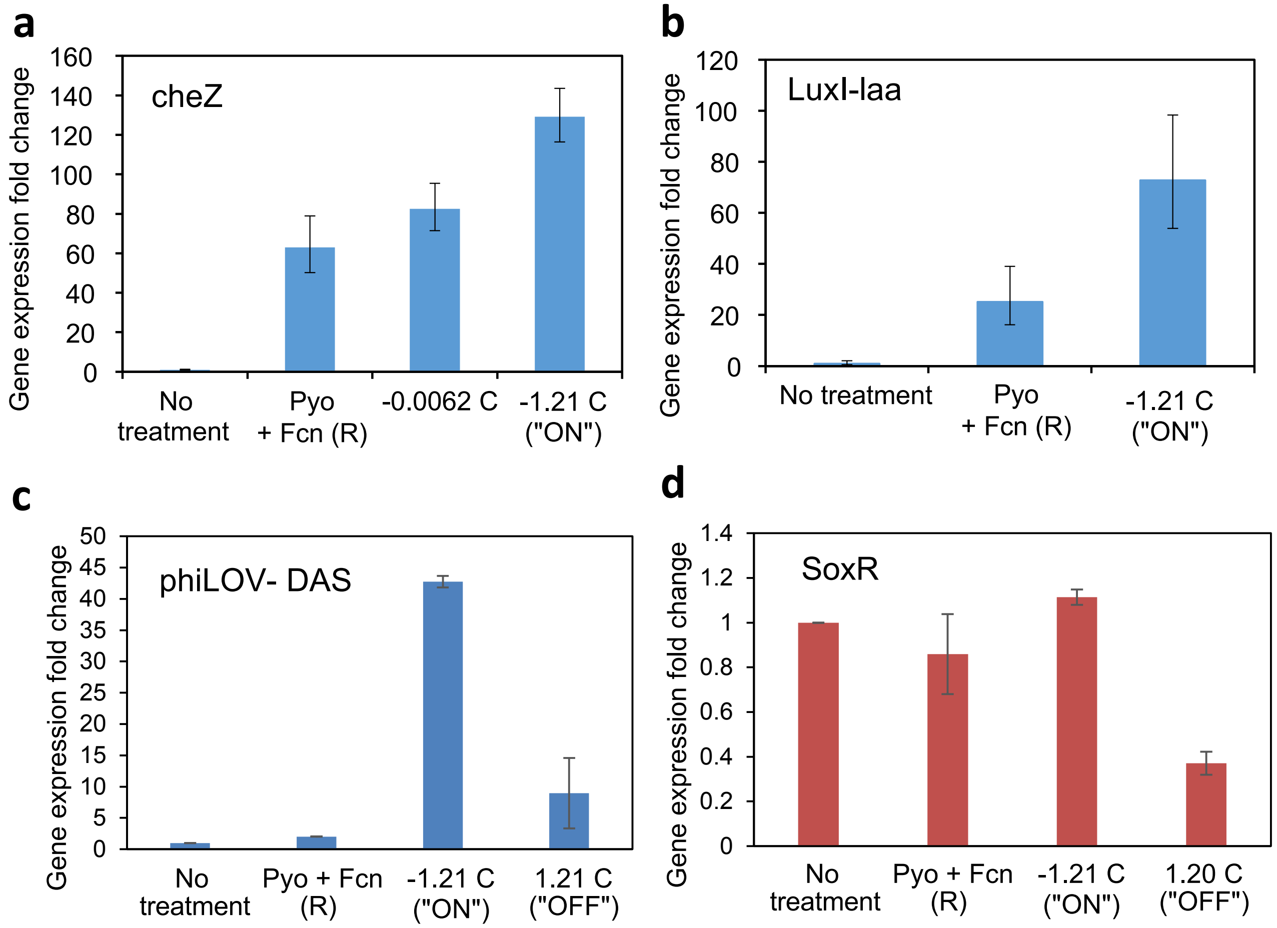

Supplementary Figure 17. qPCR analysis of electrochemically-induced cells. a. Fold change of *cheZ* upon treatment with indicated inducers and electronic induction. **b.** Fold change of *luxI* upon treatment with indicated inducers and electronic induction. **c.** Fold change of *phiLOV-DAS* upon treatment with indicated inducers and electronic induction – both ON and OFF. **d.** Fold change of *soxR* upon same treatments (and from the same samples) as in **c**. Calibrator samples are the "no treatment" samples for each gene of interest. In a and b, three technical replicates were done for one biological sample; in c and d three technical replicates were done for each of two biological replicates. Error bars plotted in a and b illustrate the range of fold change in expression based upon one standard deviation above and below the average $\Delta\Delta$Ct. In c and d error bars represent standard deviation of the two biological replicates.

Supplementary Table 1. Strains and plasmids used in this study.

Strain/Plasmid	Genotype and properties	Reference or source
Strains		
E. coli		
DJ901	GC4468 *with zjc-2205::Tn10kan Δ(soxS-soxR)566*	[1]
GC4468	*Δ(argF-lac)169 λ IN(rrnD-rrnE)1 rpsL179*(strR)	[1]
HCW01	W3110 *cheZ-*	This study
NEB5α	K-12 strain, DH5α derivative*, fhuA2 Δ(argF-lacZ)U169 phoA glnV44 Φ80 Δ(lacZ)M15 gyrA96 recA1 relA1 endA1 thi-1 hsdR17*	New England Biolabs (NEB, Ipswich, MA)
Top 10	Chemically competent, F- *mcrA* Δ(*mrr-hsd*RMS-*mcr*BC) Φ80*lac*ZΔM15 Δ *lac*X74 *rec*A1 *ara*D139 Δ(*araleu*)7697 *gal*U *gal*K *rps*L (StrR) *end*A1 *nup*G	Thermo Fisher Scientific (Waltham, MA)
W3110	K-12 strain, wild type, λ-, F-, IN(rrnD-rrnE)1, rph-1s	Genetic Stock Center Yale University (New Haven, CT)
ZK126	*Δ(argF-lac)169 λ$^-$ IN(rrnD-rrnE)1 rph-1 tnaA5*	Genetic Stock Center Yale University (New Haven, CT)
Plasmids		
707-FLPe	*flpe* driven by λR promoter and under control of a heat labile cI857 repressor, Tet^R	Gene Bridges (Heidelberg, Germany)
pBR322	Cloning vector, Ap^r, Tet^r	NEB
pCR-Blunt II-TOPO	Cloning vector, *lac* promoter, Km^r	Thermo Fisher Scientific
pFZY1	Low-copy, *lacZ* under promoter of study, Ap^r	[2]
pHW01	pFZY1-derivative, *soxR* gene and the overlapping divergent *soxR/soxS* promoters, *cheZ* downstream of *soxS* promoter, Ap^r	This work
pKD4	FRT sites flanking *kan* cassette, Apr, Km^r	[3]
pKD46	Temperature sensitive replication (repA101ts); encodes lambda Red genes (*exo*, *bet*, *gam*), Ap^r	[3]
pLuxIR2	*luxR* and *luxI* under lac/ara-1 promoter, Cm^r	[2]
pT5G	T5 promoter driving *eGFP* production, Km^r	This work
pT5RT7G	T5 promoter driving *dsRedExpress2* production, Km^r	[4,5]
pTD103Aiia	*luxR* under *luxR* promoter, *aiia* under *luxI* promoter, Ap^r	[6]
pTG1	pFZY1, *soxR* gene and the overlapping divergent P*soxR* P*soxS* promoters, *lacZ* downstream of P*soxS* promoter, Ap^r	[7]
pTT01	pBR322, *soxR* gene and the overlapping divergent P*soxR* and P*soxS* promoters, *phiLOV* downstream of P*soxS* promoter, Ap^r	This work
pTT02	pTT01 with *phiLOV-LAA*	This work
pTT03	pTT01 with *phiLOV-DAS*	This work
pTT04	pTT01 without *soxR*	This work
pTT05	pTT01 with *luxI-LAA* downstream of P*soxS*	This work
pTT06	pBR322, *luxR* under *luxR* promoter, *phiLOV* under *luxI* promoter, Ap^r	This work

Supplementary Table 2: Primers used in this work.

Gene/construct	Primers
***soxR*-P*soxS* region from MG1655 genome**	F: GGATTTGGATCCTTAGTTTTGTTCATCTTCCAGCAAG<br>R: GAACACTGAAAAGAGGCAGATTTAAGCTTAATC
***phiLOV* for Gibson**	F: GACAGCTTATCATCGATTTATTATTACACATGATCGCTGCC<br>R: GAGGCAGATTTAAGCTTATGATTGAAAAAAGCTTTGTGATTAC
**pBR322-s*oxR-PsoxS* for Gibson**	F: AAGCTTAAATCTGCCTCTTTT<br>R: ATCGATGATAAGCTGTCAAA
***phiLOV-LAA* for Gibson**	F: GACAGCTTATCATCGATTTATTATTAAGCAGCCAGAGC<br>R: CCAGTGCAGGAGCTCTTATTATTAAGCAGCCAGAGC
**DAS tag addition**	F: TAGTTTTCGTCGTTAGCAGCCAC<br>R: CGCTGACGCTTCTTAATAATAATATCGATGATAAGCTGTCAAACATGA
**pBR322-P*soxS-phiLOV-DAS***	F: TGGGAAGATGAACAAAACTAA<br>R: AAATCGCTTTACCTCAAGTTA
***luxI* gene for Gibson and –LAA tag addition**	F: GACAGCTTATCATCGATTTATTAAATTTAAGACTGCTTTTTTAAACTG<br>R: GAGGCAGATTTAAGCTTATGACTATAATGATAAAAAAATCGGATTTT<br>For tag:<br>F: TTCGTCGTTAGCAGCATTTAAGACTGCTTTTTTAAACTGTT<br>R: AACTACGCTCTGGCTGCTTAATAAATCGATGATAAGCTGTCAA
**pBR322-*soxR-PsoxS* for Gibson with *luxI***	F: AAGCTTAAATCTGCCTCTTTT<br>R: ATCGATGATAAGCTGTCAAA
***luxR-luxI* for Gibson**	F: CAAAGCTTTTTTCAATCATGGTACCTTTCTCCTCTTTAATG<br>R: TGCGTCCGGCGTAGATTAATTTTTAAAGTATGGGCAATCAATTG
**pBR322-*phiLOV* for Gibson with *luxR-luxI***	F: TCTACGCCGGACGCATCG<br>R: ATGATTGAAAAAAGCTTTGTGATTACCGATCCG
***cheZ* from W3110**	SoxS-cheZ-F: CCCCAACAGATGAATTAACGAACTGAACACTGAAAA GAGGCAGATTTATGATGCAACCATCAATCAAACCTGCTGACGAGCATTC<br>CheZ-HindIII-R: AGAGAGAGAAGCTTTCAAAATCCA AGACTATCCAAC AAATCGTCCACCTGATCCTGACTG
***soxR*-P*soxS* for CheZ plasmid (from W3110)**	BamHI-SoxR-F: CTCTCTCTGGATCCTTAGTTTTGTTCATCTTCCAGCAAG CGTGCGCC<br>SoxS-cheZ-R: GAATGCTCGTCAGCAGGTTTGATTGATGGTTGCATCA TAAATCTGCCTCTTTTCAGTGTTCAGTTCGTTAATTCATCTGTTGGGG
***cheZ* deletion, primers for kanamycin cassette**	cheZ-KO-P1F: ATGATGCAACCATCAATCAAACCTGCTGACGAGCATTCA GCTGGCGGTGTAGGCTGGAGCTGCTTC<br>cheZ-KO-P2R: TCAAAATCCAAGACTATCCAACAAATCGTCCACCTGAT CCTGACTGGCCATATGAATATCCTCCTTAG
***cheZ* deletion, primers for verification of deletion**	cheZ_ seq-P1F: CGCGTATGCTGCGCGACAGTTTGCGGGAAC<br>cheZ_seq-P2R:GCTGGTATCGACCTGAGGTCCATTAAGCAAACTCTGG
***cheZ* deletion, primers for verification of kancassette insertion**	primer set 1: cheZ-upstream: CTCCGACTGGAACATGCCCAATATG & Kt: CGGCCACAGTCGATGAATCC;<br>primer set 2: k2: CGGTGCCCTGAATGAACTGC & cheZ-downstream: CAAAGCGTTCAATGGTTTGAGTAAG

***cheZ* deletion, primers for verification of kan cassette deletion**	k1: CAGTCATAGCCGAATAGCCT<br>kt: CGGCCACAGTCGATGAATCC
**pT5G construction**	HindIIIdel-F: ATCGATGATAAGCTGTCAAACATGAGAATTAATTCTTGAAGACGAAAGGG<br>HindIIIdel-R: TTAATGCGGTAGTTTATCACAGTTAAATTGCTAACGCAGTCAGG<br>t5EGFP-F: TACACAAGAATTCATTAAAGAGGAGAAATTAACCATGGTGAGCAAGGGCGAGGAGC<br>t5EGFP-R: TTATGTAGAAGCTTGCGGCCGTTACTTGTACAGCTCGTCCATGCCGAGAGTGATC

Supplementary Table 3. Relevant genetic element sequences.

Genetic element	Sequence
**AANDENYALAA degradation tag**	GCTGCTAACGACGAAAACTACGCTCTGGCTGCT
**AANDENYADAS degradation tag**	GCTGCTAACGACGAAAACTACGCTGACGCTTCT
**phiLOV2.1 anaerobic fluorescent protein (adapted from Christie et al.[8])**	ATGATTGAAAAAAGCTTTGTGATTACCGATCCGCGCCTGCCGGATTATCCGATT<br>ATTTTTGCGAGCGATGGCTTTCTGGAACTGACCGAATATAGCCGCGAAGAAAT<br>TATGGGCCGCAACGCGCGCTTTCTGCAGGGCCCGGAAACCGATCAGGCGACCG<br>TGCAGAAAATTCGCGATGCGATTCGCGATCAGCGCGAAACCACCGTGCAGCTG<br>ATTAACTATACCAAAAGCGGCAAAAAATTTTGGAACCTGCTGCATCTGCAGCC<br>GGTGCGCGATCGCAAAGGCGGCCTGCAGTATTTTATTGGCGTGCAGCTGGTGG<br>GCAGCGATCATGTGTAA
luxI-LAA	ATGACTATAATGATAAAAAAATCGGATTTTTTGGCAATTCCATCGGAGGAGTA<br>TAAAGGTATTCTAAGTCTTCGTTATCAAGTGTTTAAGCAAAGACTTGAGTGGGA<br>CTTAGTTGTAGAAAATAACCTTGAATCAGATGAGTATGATAACTCAAATGCAG<br>AATATATTTATGCTTGTGATGATACTGAAAATGTAAGTGGATGCTGGCGTTTAT<br>TACCTACAACAGGTGATTATATGCTGAAAAGTGTTTTTCCTGAATTGCTTGGTC<br>AACAGAGTGCTCCCAAAGATCCTAATATAGTCGAATTAAGTCGTTTTGCTGTAG<br>GTAAAAATAGCTCAAAGATAAATAACTCTGCTAGTGAAATTACAATGAAACTA<br>TTTGAAGCTATATATAAACACGCTGTTAGTCAAGGTATTACAGAATATGTAAC<br>AGTAACATCAACAGCAATAGAGCGATTTTTAAAGCGTATTAAAGTTCCTTGTC<br>ATCGTATTGGAGACAAAGAAATTCATGTATTAGGTGATACTAAATCGGTTGTA<br>TTGTCTATGCCTATTAATGAACAGTTTAAAAAAGCAGTCTTAAATGCTGCTAAC<br>GACGAAAACTACGCTCTGGCTGCT TAA
***luxR* through *luxI* region**	TTAATTTTTAAAGTATGGGCAATCAATTGCTCCTGTTAAAATTGCTTTAGAAAT<br>ACTTTGGCAGCGGTTTGTTGTATTGAGTTTCATTTGCGCATTGGTTAAATGGAA<br>AGTGACAGTACGCTCACTGCAGCCTAATATTTTTGAAATATCCCAAGAGCTTTT<br>TCCTTCGCATGCCCACGCTAAACATTCTTTTTCTCTTTTGGTTAAATCGTTGTTT<br>GATTTATTATTTGCTATATTTATTTTTCGATAATTATCAACTAGAGAAGGAACA<br>ATTAATGGTATGTTCATACACGCATGTAAAAATAAACTATCTATATAGTTGTCT<br>TTTTCTGAATGTGCAAAACTAAGCATTCCGAAGCCATTGTTAGCCGTATGAATA<br>GGGAAACTAAACCCAGTGATAAGACCTGATGTTTTCGCTTCTTTAATTACATTT<br>GGAGATTTTTTATTTACAGCATTGTTTTCAAATATATTCCAATTAATTGGTGAAT<br>GATTGGAGTTAGAATAATCTACTATAGGATCATATTTTATTAAATTAGCGTCAT<br>CATAATATTGCCTCCATTTTTTAGGGTAATTATCTAGAATTGAAATATCAGATT<br>TAACCATAGAATGAGGATAAATGATCGCGAGTAAATAATATTCACAATGTACC<br>ATTTTAGTCATATCAGATAAGCATTGATTAATATCATTATTGCTTCTACAAGCT<br>TTAATTTTATTAATTATTCTGTATGTGTCGTCGGCATTTATGTTTTTCATACCCA<br>TCTCTTTATCCTTACCTATTGTTTGTCGCAAGTTTTGCGTGTTATATATCATTAA<br>AACGGTAATGGATTGACATTTGATTCTAATAAATTGGATTTTTGTCACACTATT<br>GTATCGCTGGGAATACAATTACTTAACATAAGCACCTGTAGGATCGTACAGGT<br>TTACGCAAGAAAATGGTTTGTTATAGTCGAATGAATTCATTAAAGAGGAGAAA<br>GGTACC

cheZ	ATGATGCAACCATCAATCAAACCTGCTGACGAGCATTCAGCTGGCGATATCAT<br>TGCGCGCATCGGCAGCCTGACGCGTATGCTGCGCGACAGTTTGCGGGAACTGG<br>GGCTGGATCAGGCCATTGCCGAAGCGGCGGAAGCCATCCCCGATGCGCGCGAT<br>CGTTTGTACTATGTTGTGCAGATGACCGCCCAGGCTGCGGAGCGGGCGCTGAA<br>CAGTGTTGAGGCGTCACAACCGCATCAGGATCAAATGGAGAAATCAGCAAAA<br>GCGTTAACCCAACGTTGGGATGACTGGTTTGCCGATCCGATTGACCTTGCCGAC<br>GCCCGTGAACTGGTAACAGATACACGACAATTTCTGGCAGATGTACCCGCGCA<br>TACCAGCTTTACTAACGCGCAACTGCTGGAAATCATGATGGCGCAGGATTTTC<br>AGGATCTCACCGGGCAGGTCATTAAGCGGATGATGGATGTCATTCAGGAGATC<br>GAACGCCAGTTGCTGATGGTGCTGTTGGAAAACATCCCGGAACAGGAGTCGCG<br>TCCAAAACGTGAAAACCAGAGTTTGCTTAATGGACCTCAGGTCGATACCAGCA<br>AAGCCGGTGTGGTAGCCAGTCAGGATCAGGTGGACGATTTGTTGGATAGTCTT<br>GGATTTTGA

Supplementary Table 4. Primer sequences for quantitative PCR analysis.

cheZ	F: TTGATCCTGATGCGGTTGTG<br>R: CGCGCGATCGTTTGTACTAT
phiLOV-DAS	F: AAAGCTTTGTGATTACCGATCC<br>R: GTTGCGGCCCATAATTTCTTC
luxI-LAA	F: GCAATTCCATCGGAGGAGTATAA<br>R: CTACAACTAAGTCCCACTCAAGTC
soxR	F: CGGCGCATTCATACCTTAGT<br>R: TTACGCAACGGGCAATCA

Supplementary Note 1

Increasing concentrations of pyocyanin added to the culture resulted in higher phiLOV fluorescence as measured by flow cytometry (Supplementary Fig. 4 a insert) from samples induced for 1.5 hours before fixing. The addition of 5 mM of ferricyanide increased fluorescence to a much higher degree than just pyocyanin (up to ~17-fold in the case of 5 µM of pyocyanin) (Supplementary Fig. 4 a). Increasing ferricyanide concentration also increased the fluorescence when pyocyanin was 5 µM (Supplementary Fig. 4 b). When phiLOV-DAS fluorescence was measured over time, increasing ferricyanide sustained a higher fluorescence over a longer period of time (Supplementary Figs. 4 c). Negative controls of ferrocyanide or ferricyanide alone were also tested, and as can be seen from Supplementary Figure 4 b, did not result in an increase of fluorescence. Therefore, pyocyanin and ferricyanide were both necessary for the amplified protein production (beyond pyocyanin at 5 µM) from the P*soxS* promoter, with pyocyanin initiating the protein production, and ferricyanide amplifying it.

Supplementary Note 2

Our system is concentration-dependent on both pyocyanin and ferricyanide (Supplementary Fig. 2 & 4) anaerobically. However, when oxygen is present, the effect of ferricyanide (increase of fluorescence) is either negated (in aerated conditions) or decreased (non-aerated) in the conditions tested (Supplementary Fig. 5). Fluorescence was measured after 1.5 hours of aerobic culture at 37 °C with or without 250 rpm shaking. When any pyocyanin is present, aeration allows oxygen to amplify the response to a maximum value, and the concentration of ferricyanide does not affect fluorescence at the tested conditions. Non-aerated conditions allow for some cells to not be exposed to high oxygen over the course of the experiment. This drops the Pyo and SoxR-induced fluorescence response. Ferricyanide addition recovers a maximum response due to its high concentration throughout the sample. More optimization and studies would have to be conducted in conditions with various oxygen amounts to find those in which the electrogenetic device would function non-anaerobically, but here we present preliminary studies.

Supplementary Note 3

Since ferricyanide acts as an electron acceptor in anaerobic conditions, and pyocyanin can be toxic to cells, we wanted to check the metabolic and toxicity effects of our treatments. This included the measurement of glucose consumption, acetate production, and propidium iodide staining of treated cells. To tease apart metabolic effects of our treatments vs. those due to protein production, we used both the DJ901 cells with the pTT03 plasmid and those without.

Propidium iodide staining over 9 hours of growth with the indicated mediators shows that 5 μM pyocyanin and 5 mM ferricyanide, which are the concentrations used throughout the majority of the manuscript, do not induce cell death (Supplementary Fig. 6 b). We found concentrations of ~ 50 mM Fcn (O/R) were toxic.

We measured the anaerobic consumption of glucose and production of acetate due to various treatments of both DJ901 cells with and without the pTT03 plasmid. Cells were inoculated at OD_{600} of 0.25 with the mediators in the anaerobic chamber. As can be seen from data in Supplementary Figure 6 a, whether the plasmid is present or not, adding both pyocyanin and ferricyanide to the cultures results in significantly lower production of acetate per glucose consumed. This was not the case for either ferricyanide or pyocyanin alone. Specifically, while the with-plasmid cases resulted in a slightly lower specific acetate yield on glucose than those cases without plasmid, there was no apparent influence of either redox mediator. In sum, the apparent amplification of pyocyanin-induced gene expression coincided with suppressed acetate production, but was not the cause of the suppressed acetate production (because the no-plasmid case also had low acetate production). Thus, the apparent amplification in gene expression may have been assisted by reduced metabolic byproducts, but further experimentation would be needed.

Supplementary Note 4

Supplementary Figure 8 a shows the proposed intracellular interactions. Pyocyanin oxidizes SoxR and results in phiLOV protein production. Ferricyanide interacts with the cell by acting as an electron acceptor for one or more parts of the electron transport machinery. While a well-known microbial electron acceptor, ferricyanide's exact interactions within the electron chain remain unclear [9-11].

To test whether ferricyanide specifically, by acting as a terminal electron acceptor, amplifies the production of pyocyanin-induced protein, we used an alternative electron acceptor in a similar experiment. As can be seen in Supplementary Figure 8, nitrate, a common anaerobic electron acceptor, also results in an increase in phiLOV fluorescence when pyocyanin is added to cells with pTT03. Correspondingly, nitrite, a reduced form of nitrate and a less efficient electron acceptor, does not result in as high an increase in protein production. We next used phenazine metho-sulfate (PMS) instead of pyocyanin, in similar concentrations, to induce a fluorescent response with ferricyanide addition to cells with pTT03. Supplementary Figure 8 c shows that PMS behaves similarly to pyocyanin and induces fluorescence over time in a ferricyanide-dependent manner. Importantly, PMS behaves similarly to pyocyanin when cells are electronically induced with various charges (oxidizing potential, various times, as in Methods). We see an average cell fluorescence that correlates well with the applied charge (Supplementary Figs. 8d).

These results substantiate the basic premise that Pyo-induced gene expression may be augmented due to Fcn(O)'s role as an electron acceptor and/or the involvement of electron transport mechanisms. Additional studies will need to be performed to systematically elucidate the mechanism of our electrogenetic device and the relevant redox interactions.

Supplementary Note 5

In order to separate synthesis (from promoter activity) from protein degradation, the following model was applied to the changing fluorescence levels with respect to charge: $dF/dt = r(t) - k_d F$, where F = fluorescence, $r(t)$ = rate of synthesis, and k_d = first order degradation constant. All calculations were performed using Matlab.The degradation rate of the fluorescence was measured using degradation tags in separate experiments (Supplementary Fig. 12 b). A first-order model for degradation was assumed and the exponential was fitted to the data using the Levenberg-Marquardt nonlinear least squares method. The resulting best-fit degradation constant was found (k_d = 0.0362/min).

The fluorescence data was shifted to an initial value of 0 and interpolated using piecewise cubic hermite interpolation. Combining these data with the calculated first derivatives of the interpolation, $r(t)$ was extracted. The integral of $r(t)$ was then calculated to observe the dynamics of accumulated rate with respect to charge using adaptive quadrature (ie *integral* function in Matlab). This accumulated rate (from left to right, top to bottom; increasing in positive charge) is shown in red in Supplementary Figure 13, along with the interpolation (black line) of the original data (circle). The integral is calculated up to its maximum value, which we denote "integrated protein synthesis". This terminal integrated rate is plotted with respect to the corresponding charge in Figure 3 d. The resulting relationship between the terminal integrated rate and charges produced the following fitted line $y(t) = (-1.543 * 10^5)t + 3.814$, meaning that as the charge increases, the terminal integrated rate increases linearly.
The Matlab code for the model is available upon request.

Supplementary Note 6

Solution-based induction of the control WT cells (W3110), CheZ KO cells (W3110 *cheZ*$^-$) without plasmid pHW01, and CheZ KO cells with the pHW01 plasmid were performed before measurement of CheZ protein and cell velocities. Western blots performed on WT and CheZ KO cells without pHW01 (Supplementary Fig. 14 a) show that all WT cells have CheZ and all KO cells without pHW01 show absence of CheZ regardless of the inducers used. These results demonstrate that changes in CheZ protein in the CheZ KO cells with pHW01 are due to the addition of the genetically engineered elements. Additionally, the treatments do not affect the presence (WT) or absence (CheZ KO) of CheZ. Supplementary Figure 14 c shows expanded blots of the same results as in Figure 4 b. Since we pre-incubated polyclonal antibody, additional uncharacterized bands were present.

The results in Supplementary Figure 15 show that the WT cells retain a higher relative velocity that is not affected by the mediator treatments. The CheZ KO cells that do not have pHW01 show an expected lower velocity that is not affected by mediator addition. The CheZ KO cells with pHW01 show a low velocity, similar to that of the CheZ KO cells without the plasmid when no mediators are present. Pyocyanin does not affect the velocity, but ferricyanide alone did show a slight increase. Both mediators together show a concentration-dependent

increase in velocity. These solution-based controls indicate that electronic induction as described in the paper enables CheZ induction and increased velocity relative to the non-motile knockouts.

Supplementary Note 7

The bioelectronic relay cells (DJ901 with the plasmid pTT05) and the biosensor cells (DJ901 with the plasmid pTT06) were co-cultured as described in the Methods and induced with the indicated mediators in solution to test initial effects on production of fluorescent protein by the biosensor cells. As can be seen in Supplementary Figure 16, a trend similar to when DJ901 with pTT03 cells were used is seen. That is, increasing amounts of fluorescence were measured from cells induced with Fcn (O). Pyocyanin is also needed for an amplified response. We can see that fluorescence is higher in cells that are co-cultured and induced with more negative charges (Supplementry Fig. 16 b) but that these charges are relatively closer to zero than those needed to similarly induce DJ901 cells with pTT03. This is due to LuxI & AHL's amplification of the initial Pyo and Fcn(O) signal induction, and allows for a method to tweak the sensitivity and response of the electrogenetic device.

Additionally, we performed experiments in which the relay and biosensor cells were not co-cultured. Both cells were grown as before and placed in the anaerobic chamber. The relay cells alone were electrochemically induced by oxidation of pyocyanin and ferrocyanide for various times, which resulted in the charges indicated in Supplementary Figure 16 c. After a half hour of total induction + culture time, the cells were spun down, and the supernatant filtered through a 0.22 µm filter. The supernatant was then added to the biosensor cells at OD_{600} of 0.25. These cells were then grown for 2 hours, spun down and fixed, and then fluorescence was measured with flow cytometry. As can be seen from Supplementary Figure 16 c, there is a linear correlation between the induction charge applied to the relay cells and the fluorescent response of the biosensor cells. These results indicate that an electronic induction can be translated through cell-communication molecules (AHL) into a correlated response by a second set of cells. If we can thus separate the electronic signaling and the output response while still maintaining a good correlation, we can use such bio-electronic relay cells as translators between electronics and other cells that do not have to be under anaerobic conditions or be exposed to any of the mediators.

Supplementary References

1 Greenberg, J. T., Monach, P., Chou, J. H., Josephy, P. D. & Demple, B. Positive control of a global antioxidant defense regulon activated by superoxide-generating agents in Escherichia coli. *Proc Natl Acad Sci U S A* **87**, 6181-6185, (1990).

2 You, L., Cox, R. S., 3rd, Weiss, R. & Arnold, F. H. Programmed population control by cell-cell communication and regulated killing. *Nature* **428**, 868-871, (2004).

3 Datsenko, K. A. & Wanner, B. L. One-step inactivation of chromosomal genes in Escherichia coli K-12 using PCR products. *Proc Natl Acad Sci U S A* **97**, 6640-6645, (2000).

4 Servinsky, M. D. *et al.* Directed assembly of a bacterial quorum. *The ISME journal* **10**, 158-169, (2016).

5 Wu, H. C. *et al.* Autonomous bacterial localization and gene expression based on nearby cell receptor density. *Molecular systems biology* **9**, 636, (2013).

6 Danino, T., Mondragon-Palomino, O., Tsimring, L. & Hasty, J. A synchronized quorum of genetic clocks. *Nature* **463**, 326-330, (2010).

7 Koop, A. H., Hartley, M. E. & Bourgeois, S. A low-copy-number vector utilizing beta-galactosidase for the analysis of gene control elements. *Gene* **52**, 245-256, (1987).

8 Christie, J. M. *et al.* Structural Tuning of the Fluorescent Protein iLOV for Improved Photostability. *The Journal of biological chemistry* **287**, 22295-22304, (2012).

9 Boonstra, J., Sips, H. J. & Konings, W. N. Active transport by membrane vesicles from anaerobically grown Escherichia coli energized by electron transfer to ferricyanide and chlorate. *European journal of biochemistry / FEBS* **69**, 35-44, (1976).

10 Oktyabrsky, O. N., Smirnova, G. V. & Kuznetsova, E. V. Ferricyanide reduction by Escherichia coli cells: Probable contribution of low molecular weight thiols. *Bioelectrochemistry and Bioenergetics* **32**, 267-275, (1993).

11 Hadjipetrou, L. P., Gray-Young, T. & Lilly, M. D. Effect of Ferricyanide on Energy Production by Escherichia coli. *Journal of General Microbiology* **45**, 479-488, (1966).
